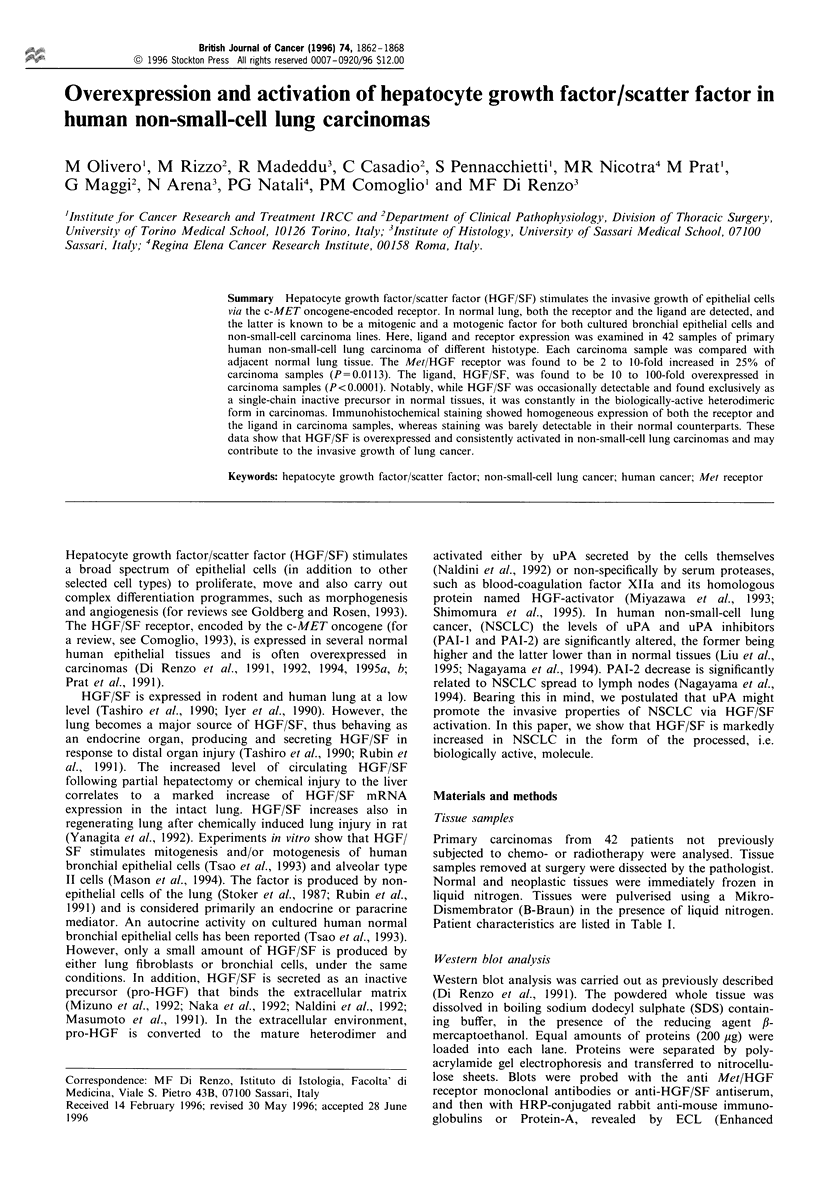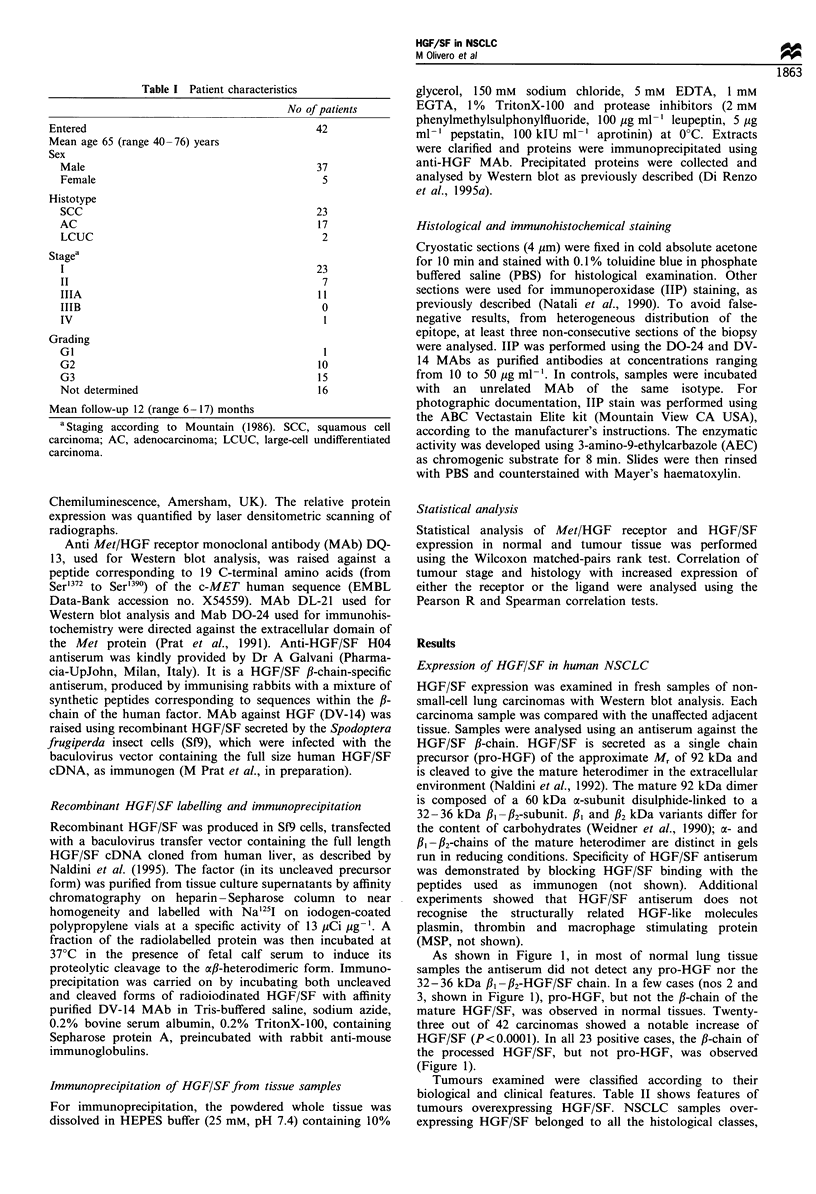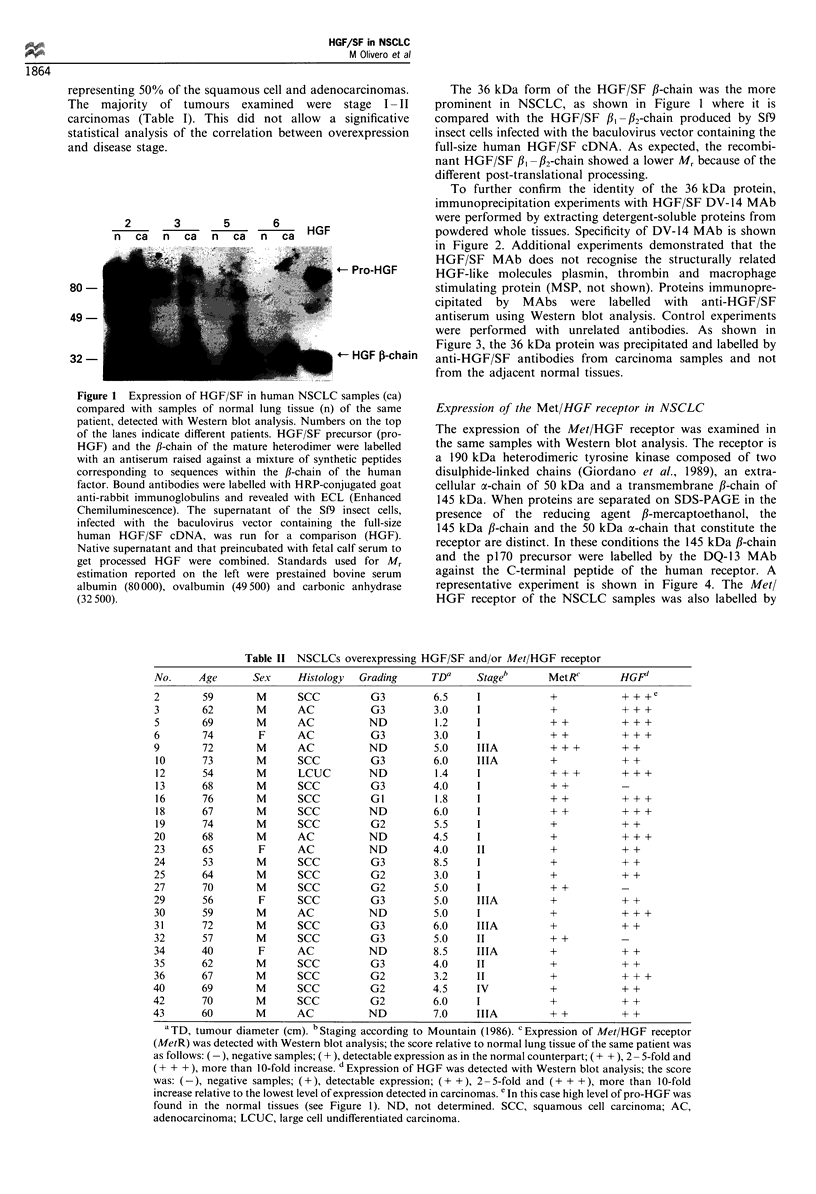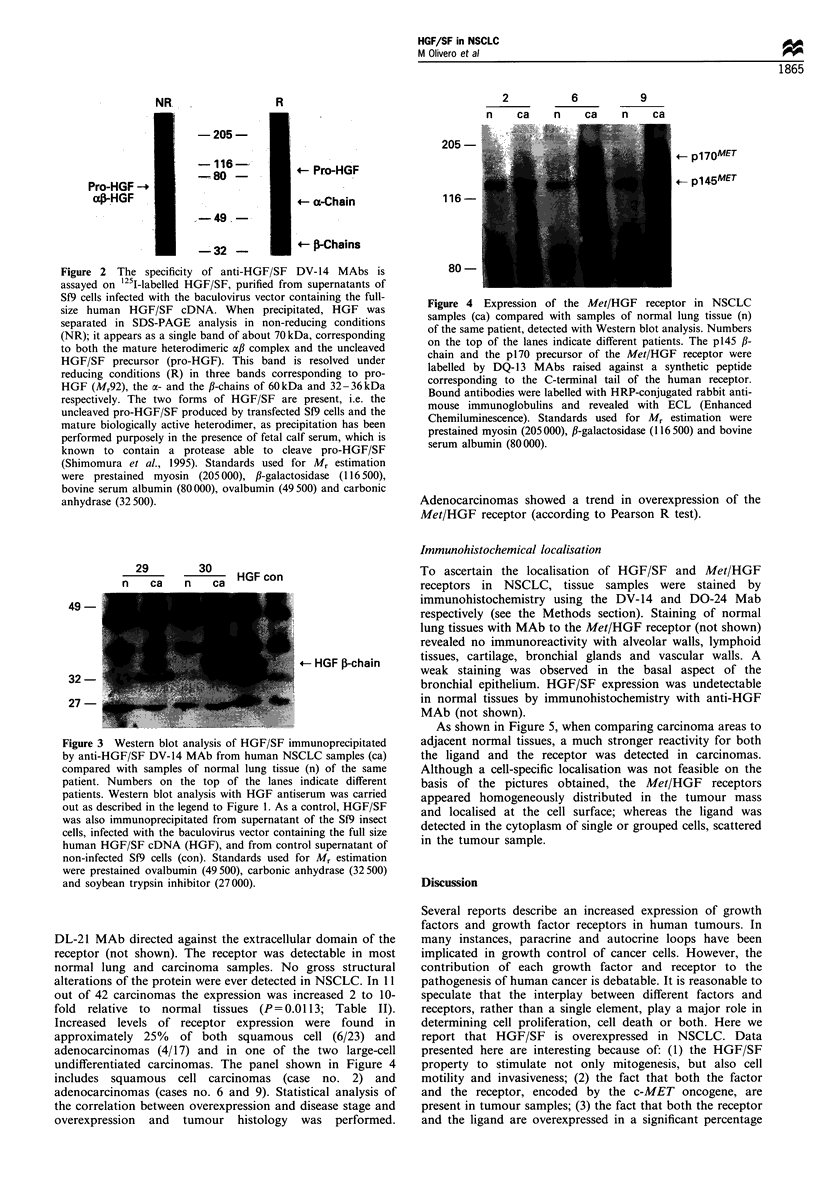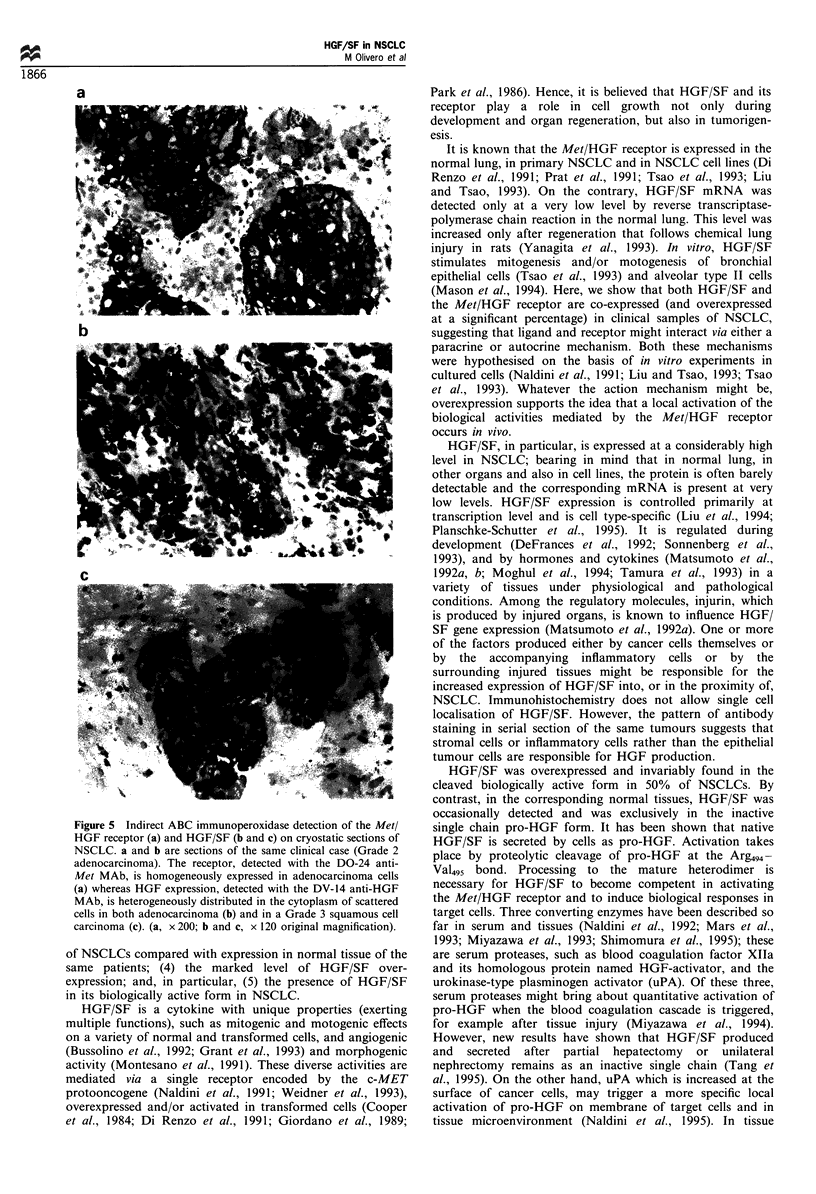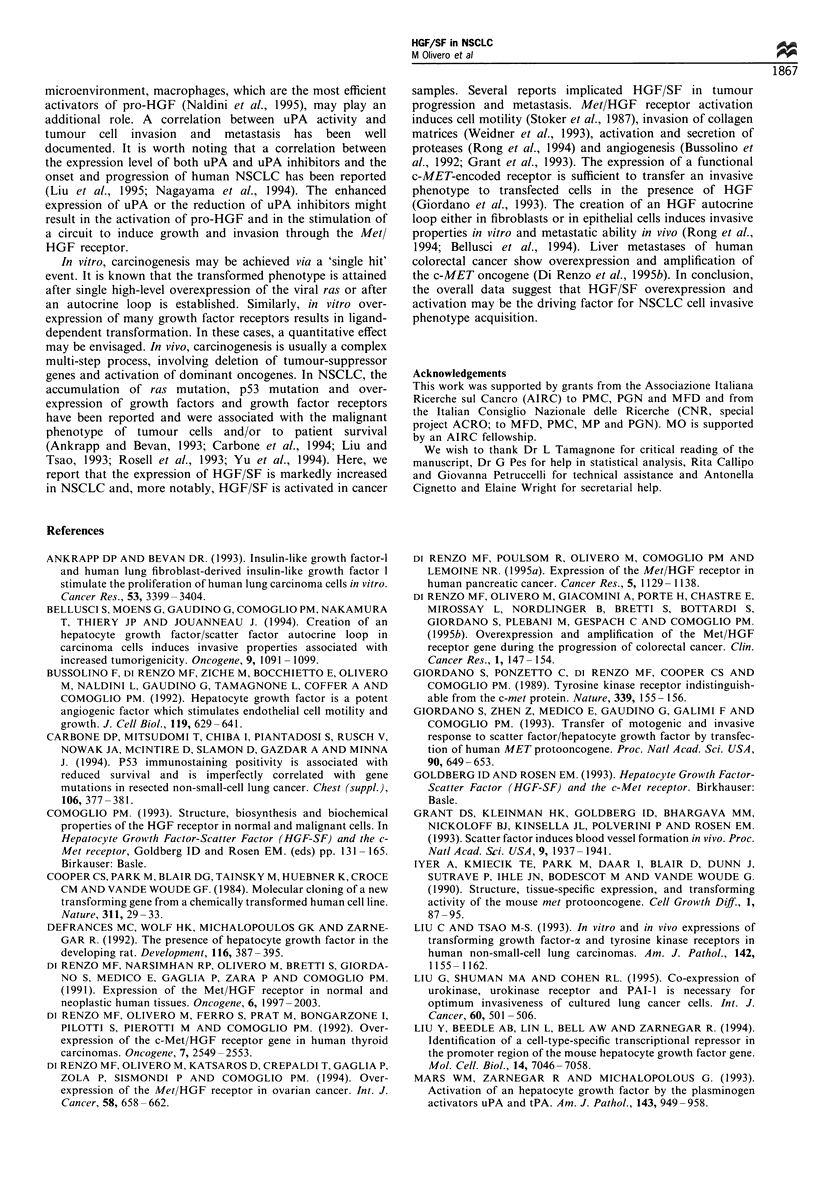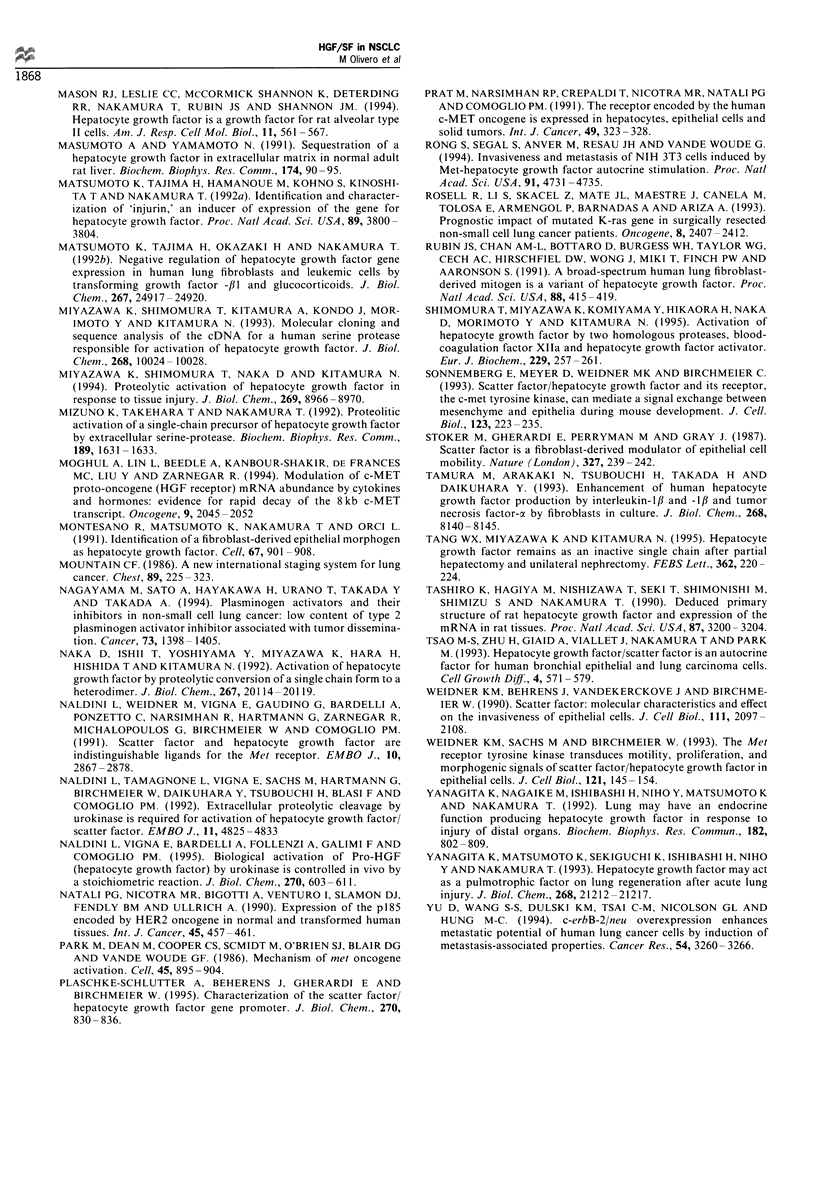# Overexpression and activation of hepatocyte growth factor/scatter factor in human non-small-cell lung carcinomas.

**DOI:** 10.1038/bjc.1996.646

**Published:** 1996-12

**Authors:** M. Olivero, M. Rizzo, R. Madeddu, C. Casadio, S. Pennacchietti, M. R. Nicotra, M. Prat, G. Maggi, N. Arena, P. G. Natali, P. M. Comoglio, M. F. Di Renzo

**Affiliations:** Institute for Cancer Research and Treatment IRCC, Torino, Italy.

## Abstract

**Images:**


British Journal of Cancer (1996) 74, 1862-1868
© 1996 Stockton Press All rights reserved 0007-0920/96 $12.00

### Overexpression and activation of hepatocyte growth factor/scatter factor in human non-small-cell lung carcinomas

M Olivero[1], M Rizzo[2], R Madeddu[3], C Casadio[2], S Pennacchietti[1], MR Nicotra[4] M Prat[1], G Maggi[2], N Arena[3], PG Natali[4], PM Comoglio[1] and MF Di Renzo[3]

*[1]Institute for Cancer Research and Treatment IRCC and [2]Department of Clinical Pathophysiology, Division of Thoracic Surgery, University of Torino Medical School, 10126 Torino, Italy; [3]Institute of Histology, University of Sassari Medical School, 07100 Sassari, Italy; [4]Regina Elena Cancer Research Institute, 00158 Roma, Italy.*

**Summary** Hepatocyte growth factor/scatter factor (HGF/SF) stimulates the invasive growth of epithelial cells *via* the c-*MET* oncogene-encoded receptor. In normal lung, both the receptor and the ligand are detected, and the latter is known to be a mitogenic and a motogenic factor for both cultured bronchial epithelial cells and non-small-cell carcinoma lines. Here, ligand and receptor expression was examined in 42 samples of primary human non-small-cell lung carcinoma of different histotype. Each carcinoma sample was compared with adjacent normal lung tissue. The *Met*/HGF receptor was found to be 2 to 10-fold increased in 25% of carcinoma samples ($P=0.0113$). The ligand, HGF/SF, was found to be 10 to 100-fold overexpressed in carcinoma samples ($P<0.0001$). Notably, while HGF/SF was occasionally detectable and found exclusively as a single-chain inactive precursor in normal tissues, it was constantly in the biologically-active heterodimeric form in carcinomas. Immunohistochemical staining showed homogeneous expression of both the receptor and the ligand in carcinoma samples, whereas staining was barely detectable in their normal counterparts. These data show that HGF/SF is overexpressed and consistently activated in non-small-cell lung carcinomas and may contribute to the invasive growth of lung cancer.

**Keywords:** hepatocyte growth factor/scatter factor; non-small-cell lung cancer; human cancer; *Met* receptor

Hepatocyte growth factor/scatter factor (HGF/SF) stimulates a broad spectrum of epithelial cells (in addition to other selected cell types) to proliferate, move and also carry out complex differentiation programmes, such as morphogenesis and angiogenesis (for reviews see Goldberg and Rosen, 1993). The HGF/SF receptor, encoded by the c-*MET* oncogene (for a review, see Comoglio, 1993), is expressed in several normal human epithelial tissues and is often overexpressed in carcinomas (Di Renzo *et al.*, 1991, 1992, 1994, 1995*a*, *b*; Prat *et al.*, 1991).

HGF/SF is expressed in rodent and human lung at a low level (Tashiro *et al.*, 1990; Iyer *et al.*, 1990). However, the lung becomes a major source of HGF/SF, thus behaving as an endocrine organ, producing and secreting HGF/SF in response to distal organ injury (Tashiro *et al.*, 1990; Rubin *et al.*, 1991). The increased level of circulating HGF/SF following partial hepatectomy or chemical injury to the liver correlates to a marked increase of HGF/SF mRNA expression in the intact lung. HGF/SF increases also in regenerating lung after chemically induced lung injury in rat (Yanagita *et al.*, 1992). Experiments *in vitro* show that HGF/SF stimulates mitogenesis and/or motogenesis of human bronchial epithelial cells (Tsao *et al.*, 1993) and alveolar type II cells (Mason *et al.*, 1994). The factor is produced by non-epithelial cells of the lung (Stoker *et al.*, 1987; Rubin *et al.*, 1991) and is considered primarily an endocrine or paracrine mediator. An autocrine activity on cultured human normal bronchial epithelial cells has been reported (Tsao *et al.*, 1993). However, only a small amount of HGF/SF is produced by either lung fibroblasts or bronchial cells, under the same conditions. In addition, HGF/SF is secreted as an inactive precursor (pro-HGF) that binds the extracellular matrix (Mizuno *et al.*, 1992; Naka *et al.*, 1992; Naldini *et al.*, 1992; Masumoto *et al.*, 1991). In the extracellular environment, pro-HGF is converted to the mature heterodimer and activated either by uPA secreted by the cells themselves (Naldini *et al.*, 1992) or non-specifically by serum proteases, such as blood-coagulation factor XIIa and its homologous protein named HGF-activator (Miyazawa *et al.*, 1993; Shimomura *et al.*, 1995). In human non-small-cell lung cancer, (NSCLC) the levels of uPA and uPA inhibitors (PAI-1 and PAI-2) are significantly altered, the former being higher and the latter lower than in normal tissues (Liu *et al.*, 1995; Nagayama *et al.*, 1994). PAI-2 decrease is significantly related to NSCLC spread to lymph nodes (Nagayama *et al.*, 1994). Bearing this in mind, we postulated that uPA might promote the invasive properties of NSCLC via HGF/SF activation. In this paper, we show that HGF/SF is markedly increased in NSCLC in the form of the processed, i.e. biologically active, molecule.

Correspondence: MF Di Renzo, Istituto di Istologia, Facolta' di Medicina, Viale S. Pietro 43B, 07100 Sassari, Italy
Received 14 February 1996; revised 30 May 1996; accepted 28 June 1996

### Materials and methods

### *Tissue samples*

Primary carcinomas from 42 patients not previously subjected to chemo- or radiotherapy were analysed. Tissue samples removed at surgery were dissected by the pathologist. Normal and neoplastic tissues were immediately frozen in liquid nitrogen. Tissues were pulverised using a Mikro-Dismembrator (B-Braun) in the presence of liquid nitrogen. Patient characteristics are listed in Table I.

### *Western blot analysis*

Western blot analysis was carried out as previously described (Di Renzo *et al.*, 1991). The powdered whole tissue was dissolved in boiling sodium dodecyl sulphate (SDS) containing buffer, in the presence of the reducing agent $\beta$-mercaptoethanol. Equal amounts of proteins (200 $\mu$g) were loaded into each lane. Proteins were separated by polyacrylamide gel electrophoresis and transferred to nitrocellulose sheets. Blots were probed with the anti *Met*/HGF receptor monoclonal antibodies or anti-HGF/SF antiserum, and then with HRP-conjugated rabbit anti-mouse immunoglobulins or Protein-A, revealed by ECL (Enhanced

Table I Patient characteristics

| | No of patients |
|---|---|
| Entered | 42 |
| Mean age 65 (range 40–76) years | |
| Sex | |
| Male | 37 |
| Female | 5 |
| Histotype | |
| SCC | 23 |
| AC | 17 |
| LCUC | 2 |
| Stage[a] | |
| I | 23 |
| II | 7 |
| IIIA | 11 |
| IIIB | 0 |
| IV | 1 |
| Grading | |
| G1 | 1 |
| G2 | 10 |
| G3 | 15 |
| Not determined | 16 |
| Mean follow-up 12 (range 6–17) months | |

[a] Staging according to Mountain (1986). SCC, squamous cell carcinoma; AC, adenocarcinoma; LCUC, large-cell undifferentiated carcinoma.

Chemiluminescence, Amersham, UK). The relative protein expression was quantified by laser densitometric scanning of radiographs.

Anti *Met*/HGF receptor monoclonal antibody (MAb) DQ-13, used for Western blot analysis, was raised against a peptide corresponding to 19 C-terminal amino acids (from $Ser^{1372}$ to $Ser^{1390}$) of the c-*MET* human sequence (EMBL Data-Bank accession no. X54559). MAb DL-21 used for Western blot analysis and Mab DO-24 used for immunohistochemistry were directed against the extracellular domain of the *Met* protein (Prat *et al.*, 1991). Anti-HGF/SF H04 antiserum was kindly provided by Dr A Galvani (Pharmacia-UpJohn, Milan, Italy). It is a HGF/SF $\beta$-chain-specific antiserum, produced by immunising rabbits with a mixture of synthetic peptides corresponding to sequences within the $\beta$-chain of the human factor. MAb against HGF (DV-14) was raised using recombinant HGF/SF secreted by the *Spodoptera frugiperda* insect cells (Sf9), which were infected with the baculovirus vector containing the full size human HGF/SF cDNA, as immunogen (M Prat *et al.*, in preparation).

### *Recombinant HGF/SF labelling and immunoprecipitation*

Recombinant HGF/SF was produced in Sf9 cells, transfected with a baculovirus transfer vector containing the full length HGF/SF cDNA cloned from human liver, as described by Naldini *et al.* (1995). The factor (in its uncleaved precursor form) was purified from tissue culture supernatants by affinity chromatography on heparin–Sepharose column to near homogeneity and labelled with $Na^{125}I$ on iodogen-coated polypropylene vials at a specific activity of 13 $\mu$Ci $\mu g^{-1}$. A fraction of the radiolabelled protein was then incubated at 37°C in the presence of fetal calf serum to induce its proteolytic cleavage to the $\alpha\beta$-heterodimeric form. Immunoprecipitation was carried on by incubating both uncleaved and cleaved forms of radioiodinated HGF/SF with affinity purified DV-14 MAb in Tris-buffered saline, sodium azide, 0.2% bovine serum albumin, 0.2% TritonX-100, containing Sepharose protein A, preincubated with rabbit anti-mouse immunoglobulins.

### *Immunoprecipitation of HGF/SF from tissue samples*

For immunoprecipitation, the powdered whole tissue was dissolved in HEPES buffer (25 mM, pH 7.4) containing 10% glycerol, 150 mM sodium chloride, 5 mM EDTA, 1 mM EGTA, 1% TritonX-100 and protease inhibitors (2 mM phenylmethylsulphonylfluoride, 100 $\mu g\ ml^{-1}$ leupeptin, 5 $\mu g\ ml^{-1}$ pepstatin, 100 kIU $ml^{-1}$ aprotinin) at 0°C. Extracts were clarified and proteins were immunoprecipitated using anti-HGF MAb. Precipitated proteins were collected and analysed by Western blot as previously described (Di Renzo *et al.*, 1995*a*).

### *Histological and immunohistochemical staining*

Cryostatic sections (4 $\mu$m) were fixed in cold absolute acetone for 10 min and stained with 0.1% toluidine blue in phosphate buffered saline (PBS) for histological examination. Other sections were used for immunoperoxidase (IIP) staining, as previously described (Natali *et al.*, 1990). To avoid false-negative results, from heterogeneous distribution of the epitope, at least three non-consecutive sections of the biopsy were analysed. IIP was performed using the DO-24 and DV-14 MAbs as purified antibodies at concentrations ranging from 10 to 50 $\mu g\ ml^{-1}$. In controls, samples were incubated with an unrelated MAb of the same isotype. For photographic documentation, IIP stain was performed using the ABC Vectastain Elite kit (Mountain View CA USA), according to the manufacturer's instructions. The enzymatic activity was developed using 3-amino-9-ethylcarbazole (AEC) as chromogenic substrate for 8 min. Slides were then rinsed with PBS and counterstained with Mayer's haematoxylin.

### *Statistical analysis*

Statistical analysis of *Met*/HGF receptor and HGF/SF expression in normal and tumour tissue was performed using the Wilcoxon matched-pairs rank test. Correlation of tumour stage and histology with increased expression of either the receptor or the ligand were analysed using the Pearson R and Spearman correlation tests.

### Results

### *Expression of HGF/SF in human NSCLC*

HGF/SF expression was examined in fresh samples of non-small-cell lung carcinomas with Western blot analysis. Each carcinoma sample was compared with the unaffected adjacent tissue. Samples were analysed using an antiserum against the HGF/SF $\beta$-chain. HGF/SF is secreted as a single chain precursor (pro-HGF) of the approximate $M_r$ of 92 kDa and is cleaved to give the mature heterodimer in the extracellular environment (Naldini *et al.*, 1992). The mature 92 kDa dimer is composed of a 60 kDa $\alpha$-subunit disulphide-linked to a 32–36 kDa $\beta_1-\beta_2$-subunit. $\beta_1$ and $\beta_2$ kDa variants differ for the content of carbohydrates (Weidner *et al.*, 1990); $\alpha$- and $\beta_1-\beta_2$-chains of the mature heterodimer are distinct in gels run in reducing conditions. Specificity of HGF/SF antiserum was demonstrated by blocking HGF/SF binding with the peptides used as immunogen (not shown). Additional experiments showed that HGF/SF antiserum does not recognise the structurally related HGF-like molecules plasmin, thrombin and macrophage stimulating protein (MSP, not shown).

As shown in Figure 1, in most of normal lung tissue samples the antiserum did not detect any pro-HGF nor the 32–36 kDa $\beta_1-\beta_2$-HGF/SF chain. In a few cases (nos 2 and 3, shown in Figure 1), pro-HGF, but not the $\beta$-chain of the mature HGF/SF, was observed in normal tissues. Twenty-three out of 42 carcinomas showed a notable increase of HGF/SF ($P<0.0001$). In all 23 positive cases, the $\beta$-chain of the processed HGF/SF, but not pro-HGF, was observed (Figure 1).

Tumours examined were classified according to their biological and clinical features. Table II shows features of tumours overexpressing HGF/SF. NSCLC samples over-expressing HGF/SF belonged to all the histological classes, representing 50% of the squamous cell and adenocarcinomas. The majority of tumours examined were stage I–II carcinomas (Table I). This did not allow a significative statistical analysis of the correlation between overexpression and disease stage.

**Figure 1** Expression of HGF/SF in human NSCLC samples (ca) compared with samples of normal lung tissue (n) of the same patient, detected with Western blot analysis. Numbers on the top of the lanes indicate different patients. HGF/SF precursor (pro-HGF) and the $\beta$-chain of the mature heterodimer were labelled with an antiserum raised against a mixture of synthetic peptides corresponding to sequences within the $\beta$-chain of the human factor. Bound antibodies were labelled with HRP-conjugated goat anti-rabbit immunoglobulins and revealed with ECL (Enhanced Chemiluminescence). The supernatant of the Sf9 insect cells, infected with the baculovirus vector containing the full-size human HGF/SF cDNA, was run for a comparison (HGF). Native supernatant and that preincubated with fetal calf serum to get processed HGF were combined. Standards used for $M_r$ estimation reported on the left were prestained bovine serum albumin (80 000), ovalbumin (49 500) and carbonic anhydrase (32 500).

The 36 kDa form of the HGF/SF $\beta$-chain was the more prominent in NSCLC, as shown in Figure 1 where it is compared with the HGF/SF $\beta_1-\beta_2$-chain produced by Sf9 insect cells infected with the baculovirus vector containing the full-size human HGF/SF cDNA. As expected, the recombinant HGF/SF $\beta_1-\beta_2$-chain showed a lower $M_r$ because of the different post-translational processing.

To further confirm the identity of the 36 kDa protein, immunoprecipitation experiments with HGF/SF DV-14 MAb were performed by extracting detergent-soluble proteins from powdered whole tissues. Specificity of DV-14 MAb is shown in Figure 2. Additional experiments demonstrated that the HGF/SF MAb does not recognise the structurally related HGF-like molecules plasmin, thrombin and macrophage stimulating protein (MSP, not shown). Proteins immunoprecipitated by MAbs were labelled with anti-HGF/SF antiserum using Western blot analysis. Control experiments were performed with unrelated antibodies. As shown in Figure 3, the 36 kDa protein was precipitated and labelled by anti-HGF/SF antibodies from carcinoma samples and not from the adjacent normal tissues.

### *Expression of the* Met/*HGF receptor in NSCLC*

The expression of the *Met*/HGF receptor was examined in the same samples with Western blot analysis. The receptor is a 190 kDa heterodimeric tyrosine kinase composed of two disulphide-linked chains (Giordano *et al.*, 1989), an extracellular $\alpha$-chain of 50 kDa and a transmembrane $\beta$-chain of 145 kDa. When proteins are separated on SDS-PAGE in the presence of the reducing agent $\beta$-mercaptoethanol, the 145 kDa $\beta$-chain and the 50 kDa $\alpha$-chain that constitute the receptor are distinct. In these conditions the 145 kDa $\beta$-chain and the p170 precursor were labelled by the DQ-13 MAb against the C-terminal peptide of the human receptor. A representative experiment is shown in Figure 4. The *Met*/HGF receptor of the NSCLC samples was also labelled by

**Table II** NSCLCs overexpressing HGF/SF and/or *Met*/HGF receptor

| *No.* | *Age* | *Sex* | *Histology* | *Grading* | *TD*[a] | *Stage*[b] | Met*R*[c] | *HGF*[d] |
|---|---|---|---|---|---|---|---|---|
| 2 | 59 | M | SCC | G3 | 6.5 | I | + | + + +[e] |
| 3 | 62 | M | AC | G3 | 3.0 | I | + | + + + |
| 5 | 69 | M | AC | ND | 1.2 | I | + + | + + + |
| 6 | 74 | F | AC | G3 | 3.0 | I | + + | + + + |
| 9 | 72 | M | AC | ND | 5.0 | IIIA | + + + | + + |
| 10 | 73 | M | SCC | G3 | 6.0 | IIIA | + | + + |
| 12 | 54 | M | LCUC | ND | 1.4 | I | + + + | + + + |
| 13 | 68 | M | SCC | G3 | 4.0 | I | + + | – |
| 16 | 76 | M | SCC | G1 | 1.8 | I | + + | + + + |
| 18 | 67 | M | SCC | ND | 6.0 | I | + + | + + + |
| 19 | 74 | M | SCC | G2 | 5.5 | I | + | + + |
| 20 | 68 | M | AC | ND | 4.5 | I | + | + + + |
| 23 | 65 | F | AC | ND | 4.0 | II | + | + + |
| 24 | 53 | M | SCC | G3 | 8.5 | I | + | + + |
| 25 | 64 | M | SCC | G2 | 3.0 | I | + | + + |
| 27 | 70 | M | SCC | G2 | 5.0 | I | + + | – |
| 29 | 56 | F | SCC | G3 | 5.0 | IIIA | + | + + |
| 30 | 59 | M | AC | ND | 5.0 | I | + | + + + |
| 31 | 72 | M | SCC | G3 | 6.0 | IIIA | + | + + |
| 32 | 57 | M | SCC | G3 | 5.0 | II | + + | – |
| 34 | 40 | F | AC | ND | 8.5 | IIIA | + | + + |
| 35 | 62 | M | SCC | G3 | 4.0 | II | + | + + |
| 36 | 67 | M | SCC | G2 | 3.2 | II | + | + + + |
| 40 | 69 | M | SCC | G2 | 4.5 | IV | + | + + |
| 42 | 70 | M | SCC | G2 | 6.0 | I | + | + + |
| 43 | 60 | M | AC | ND | 7.0 | IIIA | + + | + + |

[a]TD, tumour diameter (cm). [b]Staging according to Mountain (1986). [c]Expression of *Met*/HGF receptor (*Met*R) was detected with Western blot analysis; the score relative to normal lung tissue of the same patient was as follows: (–), negative samples; (+), detectable expression as in the normal counterpart; (+ +), 2–5-fold and (+ + +), more than 10-fold increase. [d]Expression of HGF was detected with Western blot analysis; the score was: (–), negative samples; (+), detectable expression; (+ +), 2–5-fold and (+ + +), more than 10-fold increase relative to the lowest level of expression detected in carcinomas. [e]In this case high level of pro-HGF was found in the normal tissues (see Figure 1). ND, not determined. SCC, squamous cell carcinoma; AC, adenocarcinoma; LCUC, large cell undifferentiated carcinoma.

**Figure 2** The specificity of anti-HGF/SF DV-14 MAbs is assayed on $^{125}$I-labelled HGF/SF, purified from supernatants of Sf9 cells infected with the baculovirus vector containing the full-size human HGF/SF cDNA. When precipitated, HGF was separated in SDS-PAGE analysis in non-reducing conditions (NR); it appears as a single band of about 70 kDa, corresponding to both the mature heterodimeric $\alpha\beta$ complex and the uncleaved HGF/SF precursor (pro-HGF). This band is resolved under reducing conditions (R) in three bands corresponding to pro-HGF ($M_r$92), the α- and the β-chains of 60 kDa and 32–36 kDa respectively. The two forms of HGF/SF are present, i.e. the uncleaved pro-HGF/SF produced by transfected Sf9 cells and the mature biologically active heterodimer, as precipitation has been performed purposely in the presence of fetal calf serum, which is known to contain a protease able to cleave pro-HGF/SF (Shimomura *et al.*, 1995). Standards used for $M_r$ estimation were prestained myosin (205 000), β-galactosidase (116 500), bovine serum albumin (80 000), ovalbumin (49 500) and carbonic anhydrase (32 500).

**Figure 3** Western blot analysis of HGF/SF immunoprecipitated by anti-HGF/SF DV-14 MAb from human NSCLC samples (ca) compared with samples of normal lung tissue (n) of the same patient. Numbers on the top of the lanes indicate different patients. Western blot analysis with HGF antiserum was carried out as described in the legend to Figure 1. As a control, HGF/SF was also immunoprecipitated from supernatant of the Sf9 insect cells, infected with the baculovirus vector containing the full size human HGF/SF cDNA (HGF), and from control supernatant of non-infected Sf9 cells (con). Standards used for $M_r$ estimation were prestained ovalbumin (49 500), carbonic anhydrase (32 500) and soybean trypsin inhibitor (27 000).

DL-21 MAb directed against the extracellular domain of the receptor (not shown). The receptor was detectable in most normal lung and carcinoma samples. No gross structural alterations of the protein were ever detected in NSCLC. In 11 out of 42 carcinomas the expression was increased 2 to 10-fold relative to normal tissues ($P=0.0113$; Table II). Increased levels of receptor expression were found in approximately 25% of both squamous cell (6/23) and adenocarcinomas (4/17) and in one of the two large-cell undifferentiated carcinomas. The panel shown in Figure 4 includes squamous cell carcinomas (case no. 2) and adenocarcinomas (cases no. 6 and 9). Statistical analysis of the correlation between overexpression and disease stage and overexpression and tumour histology was performed.

**Figure 4** Expression of the *Met*/HGF receptor in NSCLC samples (ca) compared with samples of normal lung tissue (n) of the same patient, detected with Western blot analysis. Numbers on the top of the lanes indicate different patients. The p145 β-chain and the p170 precursor of the *Met*/HGF receptor were labelled by DQ-13 MAbs raised against a synthetic peptide corresponding to the C-terminal tail of the human receptor. Bound antibodies were labelled with HRP-conjugated rabbit anti-mouse immunoglobulins and revealed with ECL (Enhanced Chemiluminescence). Standards used for $M_r$ estimation were prestained myosin (205 000), β-galactosidase (116 500) and bovine serum albumin (80 000).

Adenocarcinomas showed a trend in overexpression of the *Met*/HGF receptor (according to Pearson R test).

*Immunohistochemical localisation*

To ascertain the localisation of HGF/SF and *Met*/HGF receptors in NSCLC, tissue samples were stained by immunohistochemistry using the DV-14 and DO-24 Mab respectively (see the Methods section). Staining of normal lung tissues with MAb to the *Met*/HGF receptor (not shown) revealed no immunoreactivity with alveolar walls, lymphoid tissues, cartilage, bronchial glands and vascular walls. A weak staining was observed in the basal aspect of the bronchial epithelium. HGF/SF expression was undetectable in normal tissues by immunohistochemistry with anti-HGF MAb (not shown).

As shown in Figure 5, when comparing carcinoma areas to adjacent normal tissues, a much stronger reactivity for both the ligand and the receptor was detected in carcinomas. Although a cell-specific localisation was not feasible on the basis of the pictures obtained, the *Met*/HGF receptors appeared homogeneously distributed in the tumour mass and localised at the cell surface; whereas the ligand was detected in the cytoplasm of single or grouped cells, scattered in the tumour sample.

### Discussion

Several reports describe an increased expression of growth factors and growth factor receptors in human tumours. In many instances, paracrine and autocrine loops have been implicated in growth control of cancer cells. However, the contribution of each growth factor and receptor to the pathogenesis of human cancer is debatable. It is reasonable to speculate that the interplay between different factors and receptors, rather than a single element, play a major role in determining cell proliferation, cell death or both. Here we report that HGF/SF is overexpressed in NSCLC. Data presented here are interesting because of: (1) the HGF/SF property to stimulate not only mitogenesis, but also cell motility and invasiveness; (2) the fact that both the factor and the receptor, encoded by the c-*MET* oncogene, are present in tumour samples; (3) the fact that both the receptor and the ligand are overexpressed in a significant percentage

**Figure 5** Indirect ABC immunoperoxidase detection of the *Met*/HGF receptor (**a**) and HGF/SF (**b** and **c**) on cryostatic sections of NSCLC. **a** and **b** are sections of the same clinical case (Grade 2 adenocarcinoma). The receptor, detected with the DO-24 anti-*Met* MAb, is homogeneously expressed in adenocarcinoma cells (**a**) whereas HGF expression, detected with the DV-14 anti-HGF MAb, is heterogeneously distributed in the cytoplasm of scattered cells in both adenocarcinoma (**b**) and in a Grade 3 squamous cell carcinoma (**c**). (**a**, ×200; **b** and **c**, ×120 original magnification).

of NSCLCs compared with expression in normal tissue of the same patients; (4) the marked level of HGF/SF overexpression; and, in particular, (5) the presence of HGF/SF in its biologically active form in NSCLC.

HGF/SF is a cytokine with unique properties (exerting multiple functions), such as mitogenic and motogenic effects on a variety of normal and transformed cells, and angiogenic (Bussolino *et al.*, 1992; Grant *et al.*, 1993) and morphogenic activity (Montesano *et al.*, 1991). These diverse activities are mediated *via* a single receptor encoded by the c-*MET* protooncogene (Naldini *et al.*, 1991; Weidner *et al.*, 1993), overexpressed and/or activated in transformed cells (Cooper *et al.*, 1984; Di Renzo *et al.*, 1991; Giordano *et al.*, 1989; Park *et al.*, 1986). Hence, it is believed that HGF/SF and its receptor play a role in cell growth not only during development and organ regeneration, but also in tumorigenesis.

It is known that the *Met*/HGF receptor is expressed in the normal lung, in primary NSCLC and in NSCLC cell lines (Di Renzo *et al.*, 1991; Prat *et al.*, 1991; Tsao *et al.*, 1993; Liu and Tsao, 1993). On the contrary, HGF/SF mRNA was detected only at a very low level by reverse transcriptase-polymerase chain reaction in the normal lung. This level was increased only after regeneration that follows chemical lung injury in rats (Yanagita *et al.*, 1993). *In vitro*, HGF/SF stimulates mitogenesis and/or motogenesis of bronchial epithelial cells (Tsao *et al.*, 1993) and alveolar type II cells (Mason *et al.*, 1994). Here, we show that both HGF/SF and the *Met*/HGF receptor are co-expressed (and overexpressed at a significant percentage) in clinical samples of NSCLC, suggesting that ligand and receptor might interact *via* either a paracrine or autocrine mechanism. Both these mechanisms were hypothesised on the basis of *in vitro* experiments in cultured cells (Naldini *et al.*, 1991; Liu and Tsao, 1993; Tsao *et al.*, 1993). Whatever the action mechanism might be, overexpression supports the idea that a local activation of the biological activities mediated by the *Met*/HGF receptor occurs *in vivo*.

HGF/SF, in particular, is expressed at a considerably high level in NSCLC; bearing in mind that in normal lung, in other organs and also in cell lines, the protein is often barely detectable and the corresponding mRNA is present at very low levels. HGF/SF expression is controlled primarily at transcription level and is cell type-specific (Liu *et al.*, 1994; Planschke-Schutter *et al.*, 1995). It is regulated during development (DeFrances *et al.*, 1992; Sonnenberg *et al.*, 1993), and by hormones and cytokines (Matsumoto *et al.*, 1992*a*, *b*; Moghul *et al.*, 1994; Tamura *et al.*, 1993) in a variety of tissues under physiological and pathological conditions. Among the regulatory molecules, injurin, which is produced by injured organs, is known to influence HGF/SF gene expression (Matsumoto *et al.*, 1992*a*). One or more of the factors produced either by cancer cells themselves or by the accompanying inflammatory cells or by the surrounding injured tissues might be responsible for the increased expression of HGF/SF into, or in the proximity of, NSCLC. Immunohistochemistry does not allow single cell localisation of HGF/SF. However, the pattern of antibody staining in serial section of the same tumours suggests that stromal cells or inflammatory cells rather than the epithelial tumour cells are responsible for HGF production.

HGF/SF was overexpressed and invariably found in the cleaved biologically active form in 50% of NSCLCs. By contrast, in the corresponding normal tissues, HGF/SF was occasionally detected and was exclusively in the inactive single chain pro-HGF form. It has been shown that native HGF/SF is secreted by cells as pro-HGF. Activation takes place by proteolytic cleavage of pro-HGF at the $Arg_{494}$–$Val_{495}$ bond. Processing to the mature heterodimer is necessary for HGF/SF to become competent in activating the *Met*/HGF receptor and to induce biological responses in target cells. Three converting enzymes have been described so far in serum and tissues (Naldini *et al.*, 1992; Mars *et al.*, 1993; Miyazawa *et al.*, 1993; Shimomura *et al.*, 1995); these are serum proteases, such as blood coagulation factor XIIa and its homologous protein named HGF-activator, and the urokinase-type plasminogen activator (uPA). Of these three, serum proteases might bring about quantitative activation of pro-HGF when the blood coagulation cascade is triggered, for example after tissue injury (Miyazawa *et al.*, 1994). However, new results have shown that HGF/SF produced and secreted after partial hepatectomy or unilateral nephrectomy remains as an inactive single chain (Tang *et al.*, 1995). On the other hand, uPA which is increased at the surface of cancer cells, may trigger a more specific local activation of pro-HGF on membrane of target cells and in tissue microenvironment (Naldini *et al.*, 1995). In tissue microenvironment, macrophages, which are the most efficient activators of pro-HGF (Naldini *et al.*, 1995), may play an additional role. A correlation between uPA activity and tumour cell invasion and metastasis has been well documented. It is worth noting that a correlation between the expression level of both uPA and uPA inhibitors and the onset and progression of human NSCLC has been reported (Liu *et al.*, 1995; Nagayama *et al.*, 1994). The enhanced expression of uPA or the reduction of uPA inhibitors might result in the activation of pro-HGF and in the stimulation of a circuit to induce growth and invasion through the *Met*/HGF receptor.

*In vitro*, carcinogenesis may be achieved *via* a 'single hit' event. It is known that the transformed phenotype is attained after single high-level overexpression of the viral *ras* or after an autocrine loop is established. Similarly, *in vitro* overexpression of many growth factor receptors results in ligand-dependent transformation. In these cases, a quantitative effect may be envisaged. *In vivo*, carcinogenesis is usually a complex multi-step process, involving deletion of tumour-suppressor genes and activation of dominant oncogenes. In NSCLC, the accumulation of *ras* mutation, p53 mutation and overexpression of growth factors and growth factor receptors have been reported and were associated with the malignant phenotype of tumour cells and/or to patient survival (Ankrapp and Bevan, 1993; Carbone *et al.*, 1994; Liu and Tsao, 1993; Rosell *et al.*, 1993; Yu *et al.*, 1994). Here, we report that the expression of HGF/SF is markedly increased in NSCLC and, more notably, HGF/SF is activated in cancer samples. Several reports implicated HGF/SF in tumour progression and metastasis. *Met*/HGF receptor activation induces cell motility (Stoker *et al.*, 1987), invasion of collagen matrices (Weidner *et al.*, 1993), activation and secretion of proteases (Rong *et al.*, 1994) and angiogenesis (Bussolino *et al.*, 1992; Grant *et al.*, 1993). The expression of a functional c-*MET*-encoded receptor is sufficient to transfer an invasive phenotype to transfected cells in the presence of HGF (Giordano *et al.*, 1993). The creation of an HGF autocrine loop either in fibroblasts or in epithelial cells induces invasive properties *in vitro* and metastatic ability *in vivo* (Rong *et al.*, 1994; Bellusci *et al.*, 1994). Liver metastases of human colorectal cancer show overexpression and amplification of the c-*MET* oncogene (Di Renzo *et al.*, 1995*b*). In conclusion, the overall data suggest that HGF/SF overexpression and activation may be the driving factor for NSCLC cell invasive phenotype acquisition.

**Acknowledgements**
This work was supported by grants from the Associazione Italiana Ricerche sul Cancro (AIRC) to PMC, PGN and MFD and from the Italian Consiglio Nazionale delle Ricerche (CNR, special project ACRO; to MFD, PMC, MP and PGN). MO is supported by an AIRC fellowship.
We wish to thank Dr L Tamagnone for critical reading of the manuscript, Dr G Pes for help in statistical analysis, Rita Callipo and Giovanna Petruccelli for technical assistance and Antonella Cignetto and Elaine Wright for secretarial help.

### References

ANKRAPP DP AND BEVAN DR. (1993). Insulin-like growth factor-I and human lung fibroblast-derived insulin-like growth factor I stimulate the proliferation of human lung carcinoma cells *in vitro*. *Cancer Res.*, **53**, 3399–3404.

BELLUSCI S, MOENS G, GAUDINO G, COMOGLIO PM, NAKAMURA T, THIERY JP AND JOUANNEAU J. (1994). Creation of an hepatocyte growth factor/scatter factor autocrine loop in carcinoma cells induces invasive properties associated with increased tumorigenicity. *Oncogene*, **9**, 1091–1099.

BUSSOLINO F, DI RENZO MF, ZICHE M, BOCCHIETTO E, OLIVERO M, NALDINI L, GAUDINO G, TAMAGNONE L, COFFER A AND COMOGLIO PM. (1992). Hepatocyte growth factor is a potent angiogenic factor which stimulates endothelial cell motility and growth. *J. Cell Biol.*, **119**, 629–641.

CARBONE DP, MITSUDOMI T, CHIBA I, PIANTADOSI S, RUSCH V, NOWAK JA, MCINTIRE D, SLAMON D, GAZDAR A AND MINNA J. (1994). P53 immunostaining positivity is associated with reduced survival and is imperfectly correlated with gene mutations in resected non-small-cell lung cancer. *Chest (suppl.)*, **106**, 377–381.

COMOGLIO PM. (1993). Structure, biosynthesis and biochemical properties of the HGF receptor in normal and malignant cells. In *Hepatocyte Growth Factor-Scatter Factor (HGF-SF) and the c-Met receptor*, Goldberg ID and Rosen EM. (eds) pp. 131–165. Birkauser: Basle.

COOPER CS, PARK M, BLAIR DG, TAINSKY M, HUEBNER K, CROCE CM AND VANDE WOUDE GF. (1984). Molecular cloning of a new transforming gene from a chemically transformed human cell line. *Nature*, **311**, 29–33.

DEFRANCES MC, WOLF HK, MICHALOPOULOS GK AND ZARNEGAR R. (1992). The presence of hepatocyte growth factor in the developing rat. *Development*, **116**, 387–395.

DI RENZO MF, NARSIMHAN RP, OLIVERO M, BRETTI S, GIORDANO S, MEDICO E, GAGLIA P, ZARA P AND COMOGLIO PM. (1991). Expression of the Met/HGF receptor in normal and neoplastic human tissues. *Oncogene*, **6**, 1997–2003.

DI RENZO MF, OLIVERO M, FERRO S, PRAT M, BONGARZONE I, PILOTTI S, PIEROTTI M AND COMOGLIO PM. (1992). Overexpression of the c-Met/HGF receptor gene in human thyroid carcinomas. *Oncogene*, **7**, 2549–2553.

DI RENZO MF, OLIVERO M, KATSAROS D, CREPALDI T, GAGLIA P, ZOLA P, SISMONDI P AND COMOGLIO PM. (1994). Overexpression of the *Met*/HGF receptor in ovarian cancer. *Int. J. Cancer*, **58**, 658–662.

DI RENZO MF, POULSOM R, OLIVERO M, COMOGLIO PM AND LEMOINE NR. (1995*a*). Expression of the *Met*/HGF receptor in human pancreatic cancer. *Cancer Res.*, **5**, 1129–1138.

DI RENZO MF, OLIVERO M, GIACOMINI A, PORTE H, CHASTRE E, MIROSSAY L, NORDLINGER B, BRETTI S, BOTTARDI S, GIORDANO S, PLEBANI M, GESPACH C AND COMOGLIO PM. (1995*b*). Overexpression and amplification of the Met/HGF receptor gene during the progression of colorectal cancer. *Clin. Cancer Res.*, **1**, 147–154.

GIORDANO S, PONZETTO C, DI RENZO MF, COOPER CS AND COMOGLIO PM. (1989). Tyrosine kinase receptor indistinguishable from the c-*met* protein. *Nature*, **339**, 155–156.

GIORDANO S, ZHEN Z, MEDICO E, GAUDINO G, GALIMI F AND COMOGLIO PM. (1993). Transfer of motogenic and invasive response to scatter factor/hepatocyte growth factor by transfection of human *MET* protooncogene. *Proc. Natl Acad. Sci. USA*, **90**, 649–653.

GOLDBERG ID AND ROSEN EM. (1993). *Hepatocyte Growth Factor-Scatter Factor (HGF-SF) and the c-Met receptor*. Birkhauser: Basle.

GRANT DS, KLEINMAN HK, GOLDBERG ID, BHARGAVA MM, NICKOLOFF BJ, KINSELLA JL, POLVERINI P AND ROSEN EM. (1993). Scatter factor induces blood vessel formation *in vivo*. *Proc. Natl Acad. Sci. USA*, **9**, 1937–1941.

IYER A, KMIECIK TE, PARK M, DAAR I, BLAIR D, DUNN J, SUTRAVE P, IHLE JN, BODESCOT M AND VANDE WOUDE G. (1990). Structure, tissue-specific expression, and transforming activity of the mouse *met* protooncogene. *Cell Growth Diff.*, **1**, 87–95.

LIU C AND TSAO M-S. (1993). *In vitro* and *in vivo* expressions of transforming growth factor-α and tyrosine kinase receptors in human non-small-cell lung carcinomas. *Am. J. Pathol.*, **142**, 1155–1162.

LIU G, SHUMAN MA AND COHEN RL. (1995). Co-expression of urokinase, urokinase receptor and PAI-1 is necessary for optimum invasiveness of cultured lung cancer cells. *Int. J. Cancer*, **60**, 501–506.

LIU Y, BEEDLE AB, LIN L, BELL AW AND ZARNEGAR R. (1994). Identification of a cell-type-specific transcriptional repressor in the promoter region of the mouse hepatocyte growth factor gene. *Mol. Cell. Biol.*, **14**, 7046–7058.

MARS WM, ZARNEGAR R AND MICHALOPOLOUS G. (1993). Activation of an hepatocyte growth factor by the plasminogen activators uPA and tPA. *Am. J. Pathol.*, **143**, 949–958.

MASON RJ, LESLIE CC, MCCORMICK SHANNON K, DETERDING RR, NAKAMURA T, RUBIN JS AND SHANNON JM. (1994). Hepatocyte growth factor is a growth factor for rat alveolar type II cells. *Am. J. Resp. Cell Mol. Biol.*, **11**, 561–567.

MASUMOTO A AND YAMAMOTO N. (1991). Sequestration of a hepatocyte growth factor in extracellular matrix in normal adult rat liver. *Biochem. Biophys. Res. Comm.*, **174**, 90–95.

MATSUMOTO K, TAJIMA H, HAMANOUE M, KOHNO S, KINOSHITA T AND NAKAMURA T. (1992*a*). Identification and characterization of 'injurin,' an inducer of expression of the gene for hepatocyte growth factor. *Proc. Natl Acad. Sci. USA*, **89**, 3800–3804.

MATSUMOTO K, TAJIMA H, OKAZAKI H AND NAKAMURA T. (1992*b*). Negative regulation of hepatocyte growth factor gene expression in human lung fibroblasts and leukemic cells by transforming growth factor -β1 and glucocorticoids. *J. Biol. Chem.*, **267**, 24917–24920.

MIYAZAWA K, SHIMOMURA T, KITAMURA A, KONDO J, MORIMOTO Y AND KITAMURA N. (1993). Molecular cloning and sequence analysis of the cDNA for a human serine protease responsible for activation of hepatocyte growth factor. *J. Biol. Chem.*, **268**, 10024–10028.

MIYAZAWA K, SHIMOMURA T, NAKA D AND KITAMURA N. (1994). Proteolytic activation of hepatocyte growth factor in response to tissue injury. *J. Biol. Chem.*, **269**, 8966–8970.

MIZUNO K, TAKEHARA T AND NAKAMURA T. (1992). Proteolitic activation of a single-chain precursor of hepatocyte growth factor by extracellular serine-protease. *Biochem. Biophys. Res. Comm.*, **189**, 1631–1633.

MOGHUL A, LIN L, BEEDLE A, KANBOUR-SHAKIR, DE FRANCES MC, LIU Y AND ZARNEGAR R. (1994). Modulation of c-MET proto-oncogene (HGF receptor) mRNA abundance by cytokines and hormones: evidence for rapid decay of the 8 kb c-MET transcript. *Oncogene*, **9**, 2045–2052

MONTESANO R, MATSUMOTO K, NAKAMURA T AND ORCI L. (1991). Identification of a fibroblast-derived epithelial morphogen as hepatocyte growth factor. *Cell*, **67**, 901–908.

MOUNTAIN CF. (1986). A new international staging system for lung cancer. *Chest*, **89**, 225–323.

NAGAYAMA M, SATO A, HAYAKAWA H, URANO T, TAKADA Y AND TAKADA A. (1994). Plasminogen activators and their inhibitors in non-small cell lung cancer: low content of type 2 plasminogen activator inhibitor associated with tumor dissemination. *Cancer*, **73**, 1398–1405.

NAKA D, ISHII T, YOSHIYAMA Y, MIYAZAWA K, HARA H, HISHIDA T AND KITAMURA N. (1992). Activation of hepatocyte growth factor by proteolytic conversion of a single chain form to a heterodimer. *J. Biol. Chem.*, **267**, 20114–20119.

NALDINI L, WEIDNER M, VIGNA E, GAUDINO G, BARDELLI A, PONZETTO C, NARSIMHAN R, HARTMANN G, ZARNEGAR R, MICHALOPOULOS G, BIRCHMEIER W AND COMOGLIO PM. (1991). Scatter factor and hepatocyte growth factor are indistinguishable ligands for the *Met* receptor. *EMBO J.*, **10**, 2867–2878.

NALDINI L, TAMAGNONE L, VIGNA E, SACHS M, HARTMANN G, BIRCHMEIER W, DAIKUHARA Y, TSUBOUCHI H, BLASI F AND COMOGLIO PM. (1992). Extracellular proteolytic cleavage by urokinase is required for activation of hepatocyte growth factor/scatter factor. *EMBO J.*, **11**, 4825–4833

NALDINI L, VIGNA E, BARDELLI A, FOLLENZI A, GALIMI F AND COMOGLIO PM. (1995). Biological activation of Pro-HGF (hepatocyte growth factor) by urokinase is controlled in vivo by a stoichiometric reaction. *J. Biol. Chem.*, **270**, 603–611.

NATALI PG, NICOTRA MR, BIGOTTI A, VENTURO I, SLAMON DJ, FENDLY BM AND ULLRICH A. (1990). Expression of the p185 encoded by HER2 oncogene in normal and transformed human tissues. *Int. J. Cancer*, **45**, 457–461.

PARK M, DEAN M, COOPER CS, SCMIDT M, O'BRIEN SJ, BLAIR DG AND VANDE WOUDE GF. (1986). Mechanism of *met* oncogene activation. *Cell*, **45**, 895–904.

PLASCHKE-SCHLUTTER A, BEHERENS J, GHERARDI E AND BIRCHMEIER W. (1995). Characterization of the scatter factor/hepatocyte growth factor gene promoter. *J. Biol. Chem.*, **270**, 830–836.

PRAT M, NARSIMHAN RP, CREPALDI T, NICOTRA MR, NATALI PG AND COMOGLIO PM. (1991). The receptor encoded by the human c-MET oncogene is expressed in hepatocytes, epithelial cells and solid tumors. *Int. J. Cancer*, **49**, 323–328.

RONG S, SEGAL S, ANVER M, RESAU JH AND VANDE WOUDE G. (1994). Invasiveness and metastasis of NIH 3T3 cells induced by Met-hepatocyte growth factor autocrine stimulation. *Proc. Natl Acad. Sci. USA*, **91**, 4731–4735.

ROSELL R, LI S, SKACEL Z, MATE JL, MAESTRE J, CANELA M, TOLOSA E, ARMENGOL P, BARNADAS A AND ARIZA A. (1993). Prognostic impact of mutated K-ras gene in surgically resected non-small cell lung cancer patients. *Oncogene*, **8**, 2407–2412.

RUBIN JS, CHAN AM-L, BOTTARO D, BURGESS WH, TAYLOR WG, CECH AC, HIRSCHFIEL DW, WONG J, MIKI T, FINCH PW AND AARONSON S. (1991). A broad-spectrum human lung fibroblast-derived mitogen is a variant of hepatocyte growth factor. *Proc. Natl Acad. Sci. USA*, **88**, 415–419.

SHIMOMURA T, MIYAZAWA K, KOMIYAMA Y, HIKAORA H, NAKA D, MORIMOTO Y AND KITAMURA N. (1995). Activation of hepatocyte growth factor by two homologous proteases, blood-coagulation factor XIIa and hepatocyte growth factor activator. *Eur. J. Biochem.*, **229**, 257–261.

SONNEMBERG E, MEYER D, WEIDNER MK AND BIRCHMEIER C. (1993). Scatter factor/hepatocyte growth factor and its receptor, the c-met tyrosine kinase, can mediate a signal exchange between mesenchyme and epithelia during mouse development. *J. Cell. Biol.*, **123**, 223–235.

STOKER M, GHERARDI E, PERRYMAN M AND GRAY J. (1987). Scatter factor is a fibroblast-derived modulator of epithelial cell mobility. *Nature (London)*, **327**, 239–242.

TAMURA M, ARAKAKI N, TSUBOUCHI H, TAKADA H AND DAIKUHARA Y. (1993). Enhancement of human hepatocyte growth factor production by interleukin-1β and -1β and tumor necrosis factor-α by fibroblasts in culture. *J. Biol. Chem.*, **268**, 8140–8145.

TANG WX, MIYAZAWA K AND KITAMURA N. (1995). Hepatocyte growth factor remains as an inactive single chain after partial hepatectomy and unilateral nephrectomy. *FEBS Lett.*, **362**, 220–224.

TASHIRO K, HAGIYA M, NISHIZAWA T, SEKI T, SHIMONISHI M, SHIMIZU S AND NAKAMURA T. (1990). Deduced primary structure of rat hepatocyte growth factor and expression of the mRNA in rat tissues. *Proc. Natl Acad. Sci. USA*, **87**, 3200–3204.

TSAO M-S, ZHU H, GIAID A, VIALLET J, NAKAMURA T AND PARK M. (1993). Hepatocyte growth factor/scatter factor is an autocrine factor for human bronchial epithelial and lung carcinoma cells. *Cell Growth Diff.*, **4**, 571–579.

WEIDNER KM, BEHRENS J, VANDEKERCKOVE J AND BIRCHMEIER W. (1990). Scatter factor: molecular characteristics and effect on the invasiveness of epithelial cells. *J. Cell Biol.*, **111**, 2097–2108.

WEIDNER KM, SACHS M AND BIRCHMEIER W. (1993). The *Met* receptor tyrosine kinase transduces motility, proliferation, and morphogenic signals of scatter factor/hepatocyte growth factor in epithelial cells. *J. Cell Biol.*, **121**, 145–154.

YANAGITA K, NAGAIKE M, ISHIBASHI H, NIHO Y, MATSUMOTO K AND NAKAMURA T. (1992). Lung may have an endocrine function producing hepatocyte growth factor in response to injury of distal organs. *Biochem. Biophys. Res. Commun.*, **182**, 802–809.

YANAGITA K, MATSUMOTO K, SEKIGUCHI K, ISHIBASHI H, NIHO Y AND NAKAMURA T. (1993). Hepatocyte growth factor may act as a pulmotrophic factor on lung regeneration after acute lung injury. *J. Biol. Chem.*, **268**, 21212–21217.

YU D, WANG S-S, DULSKI KM, TSAI C-M, NICOLSON GL AND HUNG M-C. (1994). c-*erb*B-2/*neu* overexpression enhances metastatic potential of human lung cancer cells by induction of metastasis-associated properties. *Cancer Res.*, **54**, 3260–3266.